# Trust and Cooperation

**DOI:** 10.3389/frobt.2022.676767

**Published:** 2022-04-29

**Authors:** Benjamin Kuipers

**Affiliations:** Computer Science and Engineering, University of Michigan, Ann Arbor, MI, United States

**Keywords:** ethics, cooperation, trust, society, evolution, unknown unknowns, existential threat

## Abstract

We AI researchers are concerned about the potential impact of artificially intelligent systems on humanity. In the first half of this essay, I argue that ethics is an evolved body of cultural knowledge that (among other things) encourages individual behavior that promotes the welfare of the society (which in turn promotes the welfare of its individual members). The causal paths involved suggest that *trust* and *cooperation* play key roles in this process. In the second half of the essay, I consider whether the key role of trust exposes our society to existential threats. This possibility arises because decision-making agents (humans, AIs, and others) necessarily rely on simplified models to cope with the unbounded complexity of our physical and social world. By selecting actions to maximize a utility measure, a well-formulated game theory model can be a powerful and valuable tool. However, a poorly-formulated game theory model may be uniquely harmful, in cases where the action it recommends deliberately exploits the vulnerability and violates the trust of cooperative partners. Widespread use of such models can erode the overall levels of trust in the society. Cooperation is reduced, resources are constrained, and there is less ability to meet challenges or take advantage of opportunities. Loss of trust will affect humanity’s ability to respond to existential threats such as climate change.

## 1 Introduction and Overview

Like many researchers in Artificial Intelligence (AI), I am concerned about the impact of the increasing success of our field on the welfare of humanity. This has led many of us to look for ideas in the fields of Ethics, both philosophical and applied. And of course, to the work of anthropologists, psychologists, sociologists, historians, and others who have contributed important ideas about the roles of ethics in human society. Even in the last few years, these efforts have led to numerous books and journal articles, at least two major international conferences, and many workshops.

Although originally trained in pure mathematics, I have spent my career as an AI researcher focused on commonsense knowledge, especially cognitive maps of the spatial environment, and more generally knowledge of foundational domains (e.g., space, dynamical change, objects, actions, etc.) that help an intelligent agent make sense of its world in a computationally tractable way. This has involved reviewing literature across multiple disciplines for insights and constraints on useful representations for states of incomplete knowledge that arise during development, learning, planning, and acting.

Ethics can be viewed as another domain of foundational knowledge–a critical one at this point in time. In this essay, I describe a view from AI and robotics of certain roles that ethics plays in the welfare of humanity, and the implications of that view for how AI systems should function.

### 1.1 Terminology

This paper uses a set of terms that are familiar to many people, but which are used quite differently by different people and in different disciplines and contexts. Here are some key definitions, describing how I use these terms, followed by commentary.

An *agent* is an entity (natural or artificial) that perceives its environment, builds an internal representation, and takes actions to pursue its goals within its model of that environment [(Russell and Norvig, 2010), p.4].

A *society* is a collection of agents that share an environment and interact with each other [(Rawls, 1999), p.4]. Therefore, the environment for each agent includes the actions of other agents and their effects.

*Cooperation* is the process of two or more agents acting together for a common purpose or benefit (Tomasello et al., 2012). Coordinated individual efforts can result in greater benefits than the sum of what the individuals can accomplish (Wright, 2000; Nowak and Roger, 2011).

*Trust* is defined here as “a psychological state comprising the intention to accept vulnerability based on positive expectations of the intentions or behavior of another” [(Rousseau et al., 1998), p.1998]. This builds on a seminal model of trust (Mayer et al., 1995) that includes ability, benevolence, and integrity as three factors contributing to perceived trustworthiness.

*Ethics* ^1^ is a body of knowledge describing how a person should act in particular situations, and what sort of person one should try to be [(Shafer-Landau, 2013), p.xi]. Ethical knowledge is generally shared by members of a given society [(Tomasello, 2019), p.249].

### 1.2 Commentary

The term “agent” is used here as in the fields of artificial intelligence and multi-agent systems, encompassing both human and artificial goal-oriented actors [(Russell and Norvig, 2010), p.4]. This is not the sense of “agent” meaning someone who acts for another, the principal.

All agents, human and non-human, act to pursue goals. However, virtually all observed actions are motivated by subgoals within plans to achieve higher-level subgoals, perhaps quite distant from any ultimate goal.

In setting the foundation for his theory of justice, John Rawls writes [(Rawls, 1999), p.4] that “a society is a more or less self-sufficient association of persons who in their relations to one another recognize certain rules of conduct as binding and who for the most part act in accordance with them.”

Human agents belong to many overlapping societies, each of which may have its own ethics. The individual agent has the task of deciding what ethical knowledge applies to the current situation. The relationship between artificial agents and human societies is an important research topic.

Cooperation is a relationship among agents, which each have goals of their own, requiring the agents to resolve conflicts among individual and collective goals, as illustrated by the Prisoner’s Dilemma and other laboratory games. The collective behavior of a system of components, where the components are not “agents” capable of choosing actions to pursue their goals within the environment as they perceive it, is not considered “cooperation” by the definition used here. For example, robust distributed communication protocols such as the Internet’s TCP/IP (Cerf and Kahn, 1974) and Drone/IoT communication (Alsamhi et al., 2019) are sometimes described in terms such as “collaboration” or “cooperation” because each node in a network maintains and updates a table of accessible nodes, and the protocol selects paths for transmitting packets based on the connectivity represented by these distributed tables. Although the similarities are undeniable, we consider this case to be outside of our definition of “agent” because of the limited state and decision freedom of the nodes.

Cooperation often involves vulnerability, due to the risk of exploitation by one’s cooperative partners, who might contribute less than their share, or might take more than their share of the rewards. Therefore, voluntary cooperation requires trust of one’s partners, accepting vulnerability in the confident belief that it will not be exploited. Some cases described as “cooperation without trust” (Mayer et al., 1995) involve coerced cooperation, where credible threat of punishment eliminates risk of exploitation. Other cases (Cook et al., 2005) rely on a much stronger definition of “trust”, closer in meaning to “devoted love”, so some examples of cooperation do not involve “trust” in this strong sense.

The definition of trust above (from (Rousseau et al., 1998), inspired by (Mayer et al., 1995)) is clearly motivated by interpersonal trust between individuals who know each other, such as the trust between partners in crime in the Prisoner’s Dilemma. Of course, the word “trust” is used in many other contexts, typically with overlapping but not identical meanings. For example: trust in an attribute of an inanimate object, such as the strength of a rope, or the accuracy of a sensor; trust in the individual or corporation that manufactured or supplied that inanimate object; trust in corporate or government entities, such as the security of a savings account in a bank, or the safety and efficacy of medications allowed on the market by the FDA; trust in generic (not individually known) members of my community, such as believing that other drivers will virtually always stop at red lights, allowing me to drive confidently through a green light; and even, interpersonal trust “because we think you take our interests to heart and encapsulate our interests in your own” (Cook et al., 2005). Some of these cases are enforced by law, but it is widely recognized by legal scholars that voluntary compliance with social norms, rather than the threat of legal penalties, is primarily responsible for widespread trustworthy behavior (Posner, 2000; Posner, 2007). While these are different contexts and senses of the word “trust”, they share the social benefits described in Section 3.

In this paper, I extend the terms “cooperation” and “trust” to situations described by “social norms”, where the cooperative partners are not identified individuals, but are generic other members of the same society. For example, we trust that other drivers will stay on the correct side of the road as they drive, and will behave appropriately at stop signs and traffic lights. Near-universal obedience to these norms (and many others) makes vehicle transportation safer and more efficient for everyone involved.

Ethical knowledge is generally (though not perfectly and universally) shared by members of a given society, but it varies significantly over historical time and geographical space. Traditionally, ethical knowledge is only possessed by humans, but scholars have begun to consider how ethics applies to non-human agents such as AIs and institutions.

### 1.3 Overview: The Importance of Trust

**FIGURE 1:**
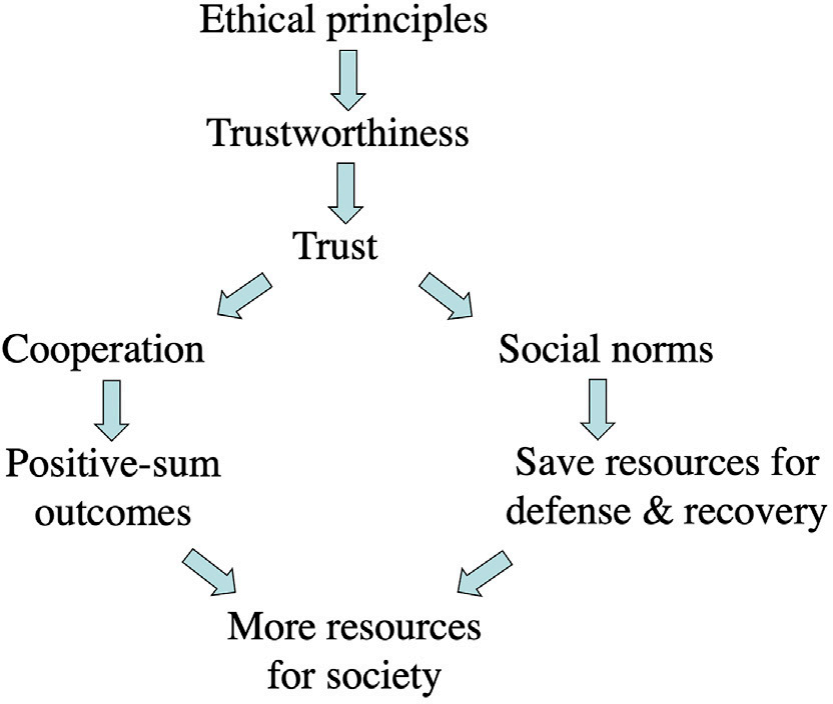
From ethics to resources.

The first half of this essay proposes a relationship among these key concepts (Figure 1), drawing on related work in philosophy (Section 2), cooperation and trust (Section 3), and evolution (Section 4).

Humanity is made up of individual humans, the agents who make decisions about how to act. Humans organize themselves into societies. Early in human evolution, societies were small isolated bands of hunter-gatherers (Tomasello, 2019). Since then, societies have grown larger, more complex, nested and overlapping in various ways. A society gets resources from the efforts of its individual members, and the individual members are supported and protected by the physical and cultural strength of the society (Wright, 2000; Christakis, 2019).

Among the assets of a society are bodies of accumulated cultural knowledge that are distributed among its individual members. This includes a great deal of “how-to” knowledge such as how to prepare specific foods and how to build specific artifacts (Henrich, 2016). The shared body of cultural knowledge also includes the *ethics* of the society, which helps to direct individuals away from possibly-tempting action choices, and toward actions that are better for the society in the long run, and therefore also better for the individual (Beauchamp and Childress, 2009; Fedyk, 2017).

We observe important similarities and striking variation in the content of the ethical knowledge in different societies, both across historical and pre-historical time, and across the different societies and cultures that exist around the world. Within a given society, knowledge is transmitted from one generation to the next through a variety of mechanisms including imitation and explicit teaching. These imperfect learning methods introduce variations, some of which fade away while others grow, persist, and displace other beliefs. The structural similarities with Darwinian evolution suggest that cultural evolution is a real and important process complementing the properties of genetic evolution (Dawkins, 1976; Richerson and Boyd, 2005; Pinker, 2011; Buchanan and Powell, 2018).

A society gets resources from the efforts of its individual members, but those efforts can be multiplied through cooperation. Mechanisms for cooperation include teamwork, specialized expertise, division of labor, economies of scale, military organization and discipline, markets, capital investments, common infrastructure, and many others. Cooperation benefits the society as a whole, as well as the individuals directly involved (Curry et al., 2016; Curry et al., 2019).

Trust and trustworthiness are widely recognized as important to the successful functioning of society (Fukuyama, 1995). A particularly important role for trust is the support of cooperation, which involves vulnerability to one’s cooperative partners. Another important role of trust is to reduce complexity and uncertainty, making it feasible to make plans by focusing on only a few possible alternatives (Luhmann, 1979; Nissenbaum, 2001).

One role of the ethical principles of a society is to help individual members of the society know how to be trustworthy, and how to recognize when others are trustworthy. Figure 1 summarizes some of the relationships among ethics, trust, cooperation, and resources for society. (This is not to argue that support for trust and cooperation are the *only* functions of ethics.)

The ethical principles of a society determine what it is to be trustworthy, and thus who or what is trusted. Trust enables cooperation which produces more resources. Trusted social norms can be counted on, saving resources. The nature and degree of trust in the society determines whether the society will have a shortage or plenty of resources, and hence whether it thrives or not in future generations.

Given the centrality of trust to the processes that provide resources for society (as shown in Figure 1), if trust is eroded, society is threatened. Lack of trust decreases both willingness to cooperate and confidence in social norms, making it harder to meet threats or exploit opportunities, resulting in scarcity of resources. As societies get larger and more complex, they increasingly rely on trust–of individuals, of institutions, and of social norms. Erosion of trust and loss of resources can bring a successful, complex society to the point of collapse (Tainter, 1988; Diamond, 2005).

### 1.4 Overview: The Vulnerability of Trust

The second half of this essay addresses the question of how trust can erode in a successful complex society.

The physical and social world we inhabit is unboundedly complex. To reason effectively, we necessarily create simplifying *models* to capture a few relevant elements of that world for current purposes, leaving all of the rest of the complexity out. Technical fields in science and engineering explicitly study the creation and evaluation of models, but simplifying models are unavoidable in everyday life and common sense as well.

A model is created for a particular purpose, and it explicitly describes a limited set of elements of the world and the relations among them. We might call these the “*known unknowns.*” We need to provide values for some of these elements in order to reason with the model, and the relationships within the model help us determine values for the others. Everything else about the infinitely complex world is treated as *negligible*–aspects of the world that we assume may be neglected for the purposes of this model. We might call these aspects the “*unknown unknowns*”.

Reasoning with incomplete knowledge–models–carries risk, but is also necessary to make it possible to draw useful conclusions. For example, reasoning about how gravity determines orbits is impossible without the simplifying “point mass assumption” that treats each body–Sun, planets, spacecraft–as if its entire mass were concentrated at a single point at its center of mass. This, of course, abstracts away geography, so that within this model, the distinction between, say, Western/European and Eastern/Asian cannot even be expressed. All this means is that one must use one model to reason about orbits, and a different one to reason about geography.

This essay presents and uses simplified, incomplete, descriptions of ethics, cooperation, trust, and evolution. Are these therefore “bad models” in the sense discussed later (in Section 7), purely by virtue of being incomplete and omitting major aspects of those topics? Not necessarily, any more than the point mass model of orbiting bodies is a bad model. After developing appropriate preliminaries, I will distinguish between harmful and useful models, drawing attention to certain types of models that may be harmful to trust in our society, leading to potentially catastrophic consequences.

To complete the astronomy analogy, suppose our goal is to predict eclipses. In the first step, a simplified model embodying the point mass assumption is used to identify precise orbits for the Earth and Moon about the Sun. The second step uses a different model, treating the Sun, Earth, and Moon as extended bodies of certain sizes and shapes (whose relative motions are now known), so we can reason about the shadows they cast and where those shadows will fall. Neither model is adequate by itself, and combining the two models is too complex, but the problem can be solved by applying one model to the first sub-problem and the other to the second.

In many cases, the simplification embodied by a model is reasonable and makes inference more efficient. But in cases where the elements omitted from the model are important, then conclusions drawn from that model may be badly wrong. The proper and improper creation and use of models is discussed in more detail in Sections 6, 7.

One dramatic example is the “Prisoner’s Dilemma”, where a straight-forward application of the powerful modeling method of game theory (von Neumann and Morgenstern, 1953; Leyton-Brown and Shoham, 2008) leads to a bad outcome due to over-simplified modeling assumptions. Another dramatic example relevant to autonomous vehicles (AVs) is the “Moral Machine” (Awad et al., 2018) where a narrowly-framed model forces a choice between two terrible evils, while a wider framing would provide a more plausible, realistic, and favorable solution. (Both are discussed in Section 7).

One possible impact of an improperly simplified model is to erode trust between potential partners and make cooperation less likely in the future. If the utility measure in a game theory model is not sensitive to trust, cooperation, or the welfare of society, then the algorithm will deliberately choose actions that exploit the vulnerabilities of other players. The overly-simple formulation of the decision model not only leads to a bad outcome, but it “poisons the well” for further decisions by discouraging trust. A generalized lack of trust can lead to inability to respond effectively to existential threats such as climate change (Section 8).

## 2 Related Work in Philosophy

### 2.1 Traditional Schools of Thought in Philosophical Ethics

Morality and ethics have been important to human society for thousands of years.

What is ethics? One philosopher responds, “*At the heart of ethics are two questions: 1) What should I do?, and 2) What sort of person should I be?*” [(Shafer-Landau, 2013), p.xi]. Another philosopher says, “*At its most basic, ethics is about … the kind of life that is most worthy of a human being, the kind of life worth choosing from among all the different ways we might live*” [(Vallor, 2016), p.2].

For centuries, moral philosophers have searched for principles to describe the moral judgments that people should make. Strong candidates include virtues (Hursthouse and Zalta, 2013), duties (Alexander et al., 2015), contractual agreements (Ashford and Mulgan, 2018; Cudd and Eftekhari, 2018), and utility maximization (Driver, 2014; Sinnott-Armstrong, 2015). No consensus has been reached.^2^ However, a repeated theme is that ethics helps balance the selfish interests of the individual decision-maker against the interests of other individuals or of the society as a whole.

Virtue ethics describes ethics in terms of the characteristic virtues of exemplary individuals, and how they confront particular problems. Aristotle (Aristotle, 1999) compares virtues to skills like carpentry, gained through experience and practice until they become automatic. A current philosopher like Shannon Vallor (Vallor, 2016) proposes “technomoral virtues” extending the traditional virtues to meet the demands of modern technological developments. The computational methods for AI knowledge representation best suited for virtue ethics are *case-based reasoning* (López, 2013) and *analogical reasoning* (Forbus et al., 2018). These methods describe specific situations in the world, actions taken, and their results and evaluations. Actions applied in past situations can be retrieved and adapted to new situations, leading to increasing experience and expertise.

Deontology is the study of duty (*deon* in Greek), which describes ethics in terms of obligations and prohibitions, offering simplicity, clarity, and ease of explanation, but raising the question of how the duties are determined. Immanuel Kant responded in 1785 with his categorical imperative: “*Act only according to that maxim whereby you can, at the same time, will that it should become a universal law*.” To apply this concept to the complexity and diversity of modern society, John Rawls (Rawls, 1999) proposed that “*The principles of justice are chosen behind a veil of ignorance*,” meaning without knowledge of the situation that one would personally occupy under those principles. The obligations and prohibitions of deontology are well suited to the expressive power of computational rules and constraints, which are standard tools for knowledge representation and inference in AI (Russell and Norvig, 2010). Isaac Asimov’s Three Laws of Robotics (Asimov, 1952) have a deontological character, but they also illustrate (through fiction) how an apparently straight-forward duty, for example “*A robot may not injure a human being or, through inaction, allow a human being to come to harm*”, can be complex and ambiguous in practical application.

Utilitarianism is the position that “*the morally right action is the action that produces the most good*” (Driver, 2014). It is a form of consequentialism, that “*the right action is understood entirely in terms of consequences produced*” (Driver, 2014). In philosophical utilitarianism, one maximizes *everyone’s* good, not just the good of the decision maker. This is in contrast with the computational methods of *game theory* (von Neumann and Morgenstern, 1953; Leyton-Brown and Shoham, 2008). On the one hand, game theory provides a powerful mathematical formalism for utilitarian calculations, including concepts of probability, discounting, and expected utility. On the other hand, the focus of game theory is on each decision-maker’s efforts to maximize their *own* utility measure (called “egoism” in (Driver, 2014)). Nonetheless, thanks to its computational power and conceptual clarity, game theory has become a near-standard for action selection in artificial intelligence and is often treated as the definition of “rationality” [(Russell and Norvig, 2010), p.611]. Recently, advocates for this “standard” view of rationality in AI have begun to reconsider their position (Russell, 2019).

### 2.2 Ethics and Artificial Intelligence

In recent decades, AI researchers have begun to create artificial entities capable of learning from data, representing knowledge, solving problems, making decisions, and taking action in our physical and social environment. Whether these entities are embodied as robots such as autonomous vehicles or are disembodied decision support systems deciding whether people get jobs, credit, or parole, they are effectively participating as members of human society.

Interest in the field of AI Ethics has grown rapidly, driven by important concerns about the impact of AI technology on human society: safety, privacy, surveillance, facial recognition, bias and fairness, polarization, etc (Christian, 2020; Kearns and Roth, 2020). Early contributions (Anderson and Anderson, 2006; Wallach and Allen, 2009; Lin et al., 2012) drew heavily on the major schools of thought in philosophical ethics.

Work in the AI Ethics research community is directed at several questions: 1) What sorts of ethical impacts are implemented AI systems likely to have on humans and human society? 2) How can AI systems be designed to make their ethical impacts on humans more positive, or at least, less negative? 3) How can we analyze and measure the impact of a particular implemented AI system on humans?

The “technomoral virtues” proposed by philosopher Shannon Vallor (Vallor, 2016) recognize that new technologies may present new and demanding ethically fraught situations requiring new (or newly framed) virtues extending the more traditional virtue ethics framework. Philosopher John Sullins (Sullins, 2020) further explores Vallor’s categories of technomoral trust and honesty, observing with concern that humans appear to have an innate tendency to trust others that can be exploited by designers of robots (Robinette et al., 2016). While humans do often exhibit initial trust, it is well known that trust can be lost and may or may not be regained. Indeed, the TIT-FOR-TAT algorithmic strategy that won two successive tournaments of the Repeated Prisoner’s Dilemma game starts with initial trust, and then responds according to the partner’s action on the previous cycle (Axelrod, 1984).

Value Sensitive Design (VSD) (Friedman et al., 2013; Friedman et al., 2021) is a general methodology for designing information systems to be compatible with human values. AI and robotic systems are embodied information systems, embedded along with humans in the physical world, so they are an important particular case for VSD methods. The concept of trust, especially for online activities, has also been analyzed by VSD researchers (Friedman et al., 2000; Nissenbaum, 2001).

Most people feel that ethical human decision-makers should be able to provide comprehensible explanations for their conclusions, and that AI decision-makers should be held to the same standard. Unfortunately, current state-of-the-art decision performance comes from deep neural network systems trained on extremely large training sets, and both their training and their operation are too complex for comprehensible explanation. This is often seen as a choice between high-performance but incomprehensible systems, vs. explainable but lower-performing systems. Wachter, et al. (Wachter et al., 2018) take a different approach, explaining the decision outcome for a given case by synthesizing artificial cases, similar to the given case, but with small differences sufficient to change the decision outcome. These “counterfactual” cases provide an explanation, not of the actual mechanism of the decision, but of the features of the case most responsible for its outcome. In a more recent paper, Mittelstadt and Wachter (Mittelstadt et al., 2019) contrast typical human styles of explanation with the model-based approaches typical in explainable-AI research. Focusing on model-based explanation of complex AI models such as deep neural networks, they discuss the limitations of simple human-comprehensible models as approximations to DNN models.

Philosophers, computer scientists, AI researchers, and experts in other areas have focused on specific aspects of AI and ethics. Computer scientist Noel Sharkey is a leader in the movement to ban killer robots (Sharkey, 2012). Philosopher Patrick Lin was among the first to propose a “Trolley Problem” analogy for autonomous vehicles (Lin, 2013), which has gone on to inspire the “Moral Machine” online survey experiment (Figure 4) (Awad et al., 2018). Some philosophers express skepticism about the relevance of ethics for robots because of supposed fundamental differences between humans and robots (van Wynsberghe and Robbins, 2019; Nyholm and Smids, 2020). Some of my own previous papers (Kuipers, 2018; Kuipers et al., 2020) explore the importance of trust to society, the appropriateness of different AI representations to ethical knowledge, and examples from several domains of what humans would want to count on from non-human agents.

Many scientific, professional, governmental, and public interest organizations in the United States, United Kingdom, and EU have formulated principles and recommendations for ethical constraints on artificial intelligence and its deployment (Cath et al., 2017). Drawing on these, the 2018 AI4People report (Floridi et al., 2018) categorizes the risks and opportunities from AI research and deployment, proposes five general principles (beneficence, non-maleficence, autonomy, justice, and explicability), the first four based on well-understood principles from applied biomedical ethics (Beauchamp and Childress, 2009). The report assumes without a definition that the reader understands the terms “trust” and “trustworthiness.” The report concludes with a list of 20 action recommendations intended to help create a “Good AI Society” based on AI technologies.

In 2019, the European Commission’s High Level Expert Group on Artificial Intelligence published its “Ethics Guidelines for Trustworthy AI” (High Level Expert Group on AI, 2019a), and in 2020 published an expanded “Assessment List for Trustworthy AI (ALTAI)” (High Level Expert Group on AI, 2020a). Two additional reports provided policy and investment recommendations (High Level Expert Group on AI, 2019b; High Level Expert Group on AI, 2020b).

These Guidelines begin with three abstract ethical principles–respect for human autonomy, prevention of harm, and fairness and explicability–plus the need to assess both benefits and risks of AI deployment, with particular attention to vulnerable groups. The Guidelines provide seven key requirements that implemented AI systems should meet: 1) human agency and oversight, 2) technical robustness and safety, 3) privacy and data governance, 4) transparency, 5) diversity, non-discrimination, and fairness, 6) environmental and societal well-being, and 7) accountability. Finally, it provides an assessment list (updated in 2020) for evaluating an implemented system.

The Guidelines provide a definition for trust in its glossary: “Trust is viewed as: 1) a set of specific beliefs dealing with benevolence, competence, integrity, and predictability (trust in beliefs); 2) the willingness of one party to depend on another in a risky situation (trusting intention); or 3) the combination of these elements” (Siau and Wang, 2018). The definition I use (Section 1) subsumes clauses (1) and (3) under a statement similar to (2), but the meanings are quite similar.

## 3 Related Work on Cooperation and Trust

Evolutionary theorists characterize *homo sapiens* as a “hyper-cooperative species,” and attribute our success as a species to the positive-sum results of cooperative action (Wright, 2000; Richerson and Boyd, 2005; Tomasello, 2019). Cooperation among individuals often yields rewards much greater than the total those individuals could obtain separately. Cooperation provides substantial advantages when faced with threats from human enemies or other predators, or when taking advantage of opportunities for obtaining more resources.

However, in a cooperative enterprise, each partner is vulnerable to exploitation by the other partners. Successful cooperation requires *trust*:

*“Trust is a psychological state comprising the intention to accept vulnerability based on positive expectations of the intentions or behavior of another.” [(* *Rousseau et al., 1998* *), p.1998]*

Where that trust exists, cooperation is possible, the society benefits from more positive-sum (“win-win”) interactions, and it tends to grow in resources. Where that trust does not exist, cooperation is much less viable, interactions are more often zero-sum or negative-sum, and the society tends to lose resources. Fewer resources, and decreased ability to mount a cooperative response to a crisis (external attack, ecological failure, epidemic disease, climate change, etc.), means that a society that once could surmount a crisis through cooperative action, no longer can, and may collapse (Tainter, 1988; Diamond, 2005).

A society has its own set of norms that show its individual members how to act in order to be considered trustworthy (Posner, 2000). They also show what sorts of behavior by others provides evidence that they are (or are not) trustworthy. Some norms, such as prohibitions against killing, stealing, breaking promises, or driving on the wrong side of the road, provide direct benefits in terms of safety. Other norms, like customs in clothing, speech, and table manners, signal that one belongs to a particular society. The presumption that in-group members are more likely to be trustworthy, while out-group members are less likely to be, encourages trust and cooperation among members of the society. However, this mechanism also encourages discrimination and racism against non-members (Posner, 2000; Van Bavel and Packer, 2021).

A contrary argument by Cook, Hardin and Levi in “*Cooperation Without Trust*?” (Cook et al., 2005) depends on a restrictive definition of trust: “According to this conception of trust, we trust you because we think you take our interests to heart and encapsulate our interests in your own. …. By ‘encapsulate’ we mean that to some extent our interests become yours in the trust relation between us” [(Cook et al., 2005), p.5]. Further: “Note that the conception of trust as encapsulated interest implies that *many interactions in which there is successful coordination or cooperation do not actually involve trust*.” [(Cook et al., 2005), p.8, emphasis theirs]. Under the broader definition cited above, the acceptance of vulnerability necessary for cooperation does require trust.

### 3.1 Is Ethics Only for Cooperation?

Anthropologist Oliver Scott Curry and his colleagues present a theory, “Morality as Cooperation” (MAC) (Curry et al., 2016; Curry et al., 2019), arguing that “morality consists of a collection of biological and cultural solutions to the problems of cooperation recurrent in human social life” [(Curry et al., 2019), p.48].

Curry and others quote an array of philosophers back to Plato and Aristotle in support of the strong connection between morality and cooperation and the common good. Based on evolutionary biology and game theory, they describe seven different problems of cooperation: 1) the allocation of resources to kin; 2) coordination to mutual advantage; 3) social exchange; 4) hawkish and 5) dovish displays of traits for resolving conflicts; 6) division; and 7) possession. Cooperative solutions to these problems explain corresponding types of morality: 1) family values; 2) group loyalty; 3) reciprocity; 4) bravery; 5) respect; 6) fairness; and 7) property rights.

Curry, Mullins, and Whitehouse (Curry et al., 2019) describe the predictions of the MAC theory for what should be considered good or bad in particular cultures, and present the results of testing those predictions against 60 societies studied by anthropologists and described in the Human Relations Area File (HRAF). They found that the predicted cooperative behaviors were almost always noted in the HRAF description, and that the descriptions were uniformly positive.

Although they make a strong case for a link from ethics to the welfare of society via cooperation, Curry et al. (Curry et al., 2019) deliberately and explicitly fall into a trap that philosopher Allen Buchanan calls the Cooperation Dogma: the claim that morality is *nothing but* a mechanism for encouraging cooperation [(Buchanan, 2020), pp.12–14]. Such a strong claim invites falsification by examples of issues that are clearly moral, but that are not about cooperation. Critics of the Cooperation Dogma present a variety of phenomena, including disgust reactions, sexual practices, the treatment of dead bodies, and the treatment of cattle in India, to argue against the “*nothing but*” claim [(Curry et al., 2019), Comments].

Buchanan makes a more limited point:

*“I cheerfully acknowledge that moralities originally were all about cooperation, and that moralities remain essential for successful cooperation today and always will be. I also heartily endorse the hypothesis that the basic features of human moral psychology, the moral mind, came about through natural selection because they contributed to cooperation and thereby to reproductive fitness. Nevertheless, I will argue that some moralities are more than a collection of solutions to cooperation problems.” [(* *Buchanan, 2020* *), p.13, his emphasis]*

Buchanan’s cheerful acknowledgment and hearty endorsement suggest that the role of trust might be part of a more nuanced understanding of the purpose of ethics.

### 3.2 Roles for Trust

My claim in this essay is that trustworthiness, and therefore properly earned trust, are key steps on the path from ethics to a thriving society via cooperation (Figure 1). With adequate trust, individuals can cooperate, producing (on average) outcomes with net positive gains for the society as a whole. When people can be trusted (most of the time) to follow social norms, then individuals can count on those social norms when they make their plans and act to achieve their goals.

Some norms (e.g., “*Keep your promises*”) are obviously important for cooperation. Other norms (e.g., “*Drive on the correct side of the road*”) are conventional, but if everyone can count on them, everyone’s travel becomes safer and more efficient. Yet others (e.g., “*Wear business attire when doing this job*”) are also conventional and seem to have little to do with cooperation, but signal membership in some group, providing evidence for trustworthiness.

Some moral principles (e.g., “*Care for elderly and disabled members of your community*” or “*Care for the dead bodies of your fallen comrades*”) explicitly direct resources toward individuals who cannot contribute productively to the society. However, they are clearly grounded in trust, by the members of a community, that their community will continue to support them even when they are unable to contribute. That trust supports risky types of cooperation, for example, participation in dangerous hunts or warfare. Similarly, trust enables commitments that accept lifelong opportunity costs in order to benefit society, for example raising children, devotion to a religious vocation, or academic pursuit and conveyance of knowledge.

Trust also provides practical benefits for the computational complexity of reasoning about the effects of actions on the world. Philosopher Helen Nissenbaum (Nissenbaum, 2001) describes important insights about the function of trust from the social theorist Niklas Luhmann (Luhmann, 1979).

*“Luhman characterizes trust as a mechanism that reduces complexity and enables people to cope with the high levels of uncertainty and complexity of contemporary life. Trust makes uncertainty and complexity tolerable because it enables us to focus on only a few possible alternatives. Humans, if faced with a full range of alternatives, if forced to acknowledge and calculate all possible outcomes of all possible decision nodes, would freeze in uncertainty and indecision. In this state, we might never be able to act in situations that call for action and decisiveness. In trusting, Luhmann says, ‘one engages in an action as though there were only certain possibilities in the future.’ Trust also enables ‘co-operative action and individual but coordinated action: trust, by the reduction of complexity, discloses possibilities for action which would have remained improbable and unattractive without trust—which would not, in other words, have been pursued.’ According to this account, trust expands people’s capacity to relate successfully to a world whose complexity, in reality, is far greater than we are capable of taking in.” [(* *Nissenbaum, 2001* *), p.106 (footnotes omitted)]*

The observations in this section support the structure described in Figure 1 connecting ethics to trust to cooperation–both explicit cooperation with selected partners and implicit cooperation through social norms–leading to regularities that one can count on, and thus to a safer, more prosperous, and more secure society.

## 4 Related Work on Evolution

The ethical principles of societies around the world, and across historical and pre-historical time, have much in common, but there are also dramatic differences. This pattern of diversity, changing over time, suggests the results of an evolutionary process. Since the ethics of a society consists of shared knowledge, that evolutionary process must operate at a cultural level, as well as (perhaps) at a biological level.

**FIGURE 2:**
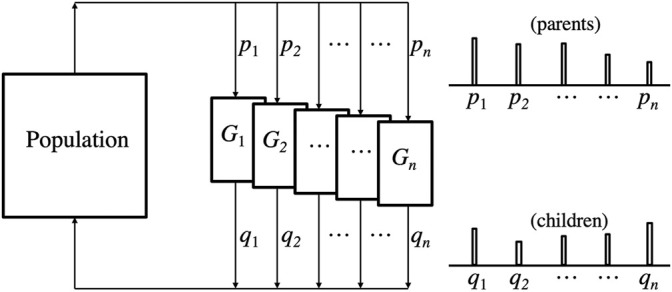
A simple sketch of biological evolution. Consider a population of individuals who can be described as having certain *genotypes*, *G*_1_, … , *G*_*n*_ with proportions *p*_1_, … , *p*_*n*_. The genotype of each individual determines its *phenotype* (not shown) which determines the proportions *q*_1_, … , *q*_*n*_, of the genotypes surviving into the population of the next generation. The two histograms (parents and children) illustrate distributional change from one generation to the next. Over many generations, new genotypes may become dominant, while others become rare or disappear entirely.

Figure 2 illustrates how biological evolution incrementally changes the distribution of genotypes in a population from one generation to the next. Over extended time, these incremental shifts can result in major qualitative changes. This pattern can be generalized to describe the accumulation and change of cultural knowledge, including ethics (Richerson and Boyd, 2005; Henrich, 2016).

One selective pressure on the ethical beliefs of a society is the ability of that society to engage in cooperative activities that increase its resources and security. To accomplish this, a society must encourage its individual members to trust each other and the institutions of the society. This evolutionary process has a number of related aspects.

### 4.1 The Evolution of Shared Intentionality

An important cognitive skill is the ability to understand the behavior of oneself or others as *agents*; that is, in terms of *actions* taken in particular *situations* to pursue one’s *goals*. Knowledge of any two of these provides some degree of information about the third.

*Observing an agent’s actions, predict its goals.*

*Knowing an agent’s goals, predict its actions in a given situation.*

*Knowing an agent’s goals and observing its actions, predict its beliefs about the current situation.*

From an evolutionary perspective, it is obviously of great value for an agent to have the ability to predict the goals, beliefs, and actions of other agents, whether they are potential cooperative partners, enemies, or prey. Michael Tomasello calls this capability, shared by humans, great apes, and other animals, *individual intentionality* (Tomasello, 2019).

Based on data from similar tests administered to chimpanzees, orangutans, and human two-and-a-half-year-old children, Tomasello’s group found strong similarities between great apes and human children in physical cognition (e.g., space, objects, and causality), and dramatic differences in social cognition and cooperation (Herrmann et al., 2007). Tomasello explains the extraordinary levels of cooperation seen in *Homo sapiens* in terms of two distinct levels of *shared intentionality*, rarely observed in non-human animals.

*“In this view, humans’ abilities to cooperate with one another take unique forms because individuals are able to create with one another a shared agent “we”, operating with shared intentions, shared knowledge, and shared sociomoral values. The claim is that these abilities emerged first in human evolution between collaborative partners operating dyadically in acts of *joint intentionality*, and then later among individuals as members of a cultural group in acts of *collective intentionality*.” [(* *Tomasello, 2019* *), p.7, emphasis added]*

Tomasello argues that joint and collective intentionality are genetically encoded biological capabilities, acquired by the species through natural selection. *Joint intentionality* appeared about 400,000 years ago (in *Homo heidelbergensis*), driven by climate change, which made food harder to come by. Humans able to cooperate with partners, for example to capture larger animals, had a competitive advantage over those who could only seek food as individuals. Likewise, the ability to cooperate pairwise in the raising of young children would be a selective advantage.

He argues that *collective intentionality* appeared around 100,000 years ago (in *Homo sapiens*), driven by increasing human population and increasing competition among human groups. Those capable of organizing into bands or tribes for collective support and defense would have an important advantage. Individuals in such groups who were incapable of learning and following the group’s social norms would face exclusion and death.

Darwin, in *The Descent of Man* (Darwin, 1874), recognized this selective pressure.

*“When two tribes of primeval man, living in the same country, came into competition, if (other circumstances being equal) the one tribe included a great number of courageous, sympathetic and faithful members, who were always ready to warn each other of danger, to aid and defend each other, this tribe would succeed better and conquer the other. …. A tribe rich in the above qualities would spread and be victorious over other tribes; but in turn overcome by some other tribe still more highly endowed.”*

The “we” underlying joint intentionality is an abstract “agent” representing the shared intention, the shared understanding of the situation, and the roles in the shared activity. For example, a joint intention to hunt antelope might have roles for the chaser and the spearer. Each individual in the dyad has an obligation to the joint “we” to fill one of the roles, and the right to share in the rewards of the joint action.

In collective intentionality, the abstract agent “we” represents the entire community, and defines rights and obligations for members of the community. Some of these obligations are norms specifying the expected behavior of members in good standing of the society, including behaving in trustworthy ways when cooperating with others within the society. Other norms may define seemingly arbitrary behavioral regularities (e.g., of dress, food, and language, etc.) that signal membership in a specific society, allowing other members to distinguish “insiders” from “outsiders” even when the society is too large to recognize everyone individually. Social psychologists Jay Van Bavel and Dominic Packer (Van Bavel and Packer, 2021) describe the positive and negative impacts of these group-based identities on individuals and societies.

The abilities to reason about individual, joint, and collective intentionality are closely related to “Theory of Mind” in child development (Gopnik and Wellman, 1992; Wellman, 2014).

*“Mirroring the phylogenetic sequence, this maturational process unfolds in two basic steps: first is the emergence of joint intentionality at around nine months of age, and second is the emergence of collective intentionality at around three years of age.” [(* *Tomasello, 2019* *), p.8]*

Parents invest substantial effort in teaching these skills and social norms to their children, since survival may depend on them.

### 4.2 The Evolution of Cultural Knowledge

Biological evolution through natural selection of genes that enhance successful reproduction is a slow process. This is plausible for the genetic evolution of the biological (neural) capacity for joint and collective intentionality over several hundred thousand years. However, the last 10,000 years or so has seen dramatic changes in the structure of our civilization, in part due to changes in the nature and scope of cooperation (Wright, 2000). These rapid changes suggest a process of cultural evolution operating at a faster time-scale.

Richerson and Boyd (Richerson and Boyd, 2005) argue that cultural evolution is a distinct process within the framework of Darwinian evolution.

*“Culture is information capable of affecting individuals’ behavior that they acquire from other members of their species through teaching, imitation, and other forms of social transmission.” [(* *Richerson and Boyd, 2005* *), p.5]*

*“Some beliefs make people more likely to be imitated, because the people who hold those beliefs are more likely to survive or more likely to achieve social prominence. Such beliefs will tend to spread, while beliefs that lead to early death or social stigma will disappear.” [(* *Richerson and Boyd, 2005* *), p.6]*

*“ …the human cultural system arose as an adaptation, because it can evolve fancy adaptations to changing environments rather more swiftly than is possible by genes alone. Culture would never have evolved unless it could do things that genes can’t.” [(* *Richerson and Boyd, 2005* *), p.7]*

It is important to recognize that cultural evolution is a kind of evolution by natural selection, but the analogy with biological evolution is not comprehensive. New variations are not generated through random mutations, but through inspiration or errors by individual humans. They are not selected purely through differential survival and reproduction, but by ease and accuracy of transmission of ideas from some human minds to others (Dawkins, 1976).

Joseph Henrich, in *The Secret of Our Success* (Henrich, 2016), sets out to explain the unique dominance of *homo sapiens* over the other species on our planet. Even before the beginning of recorded history, early humans had settled over a larger and more diverse geographical range than any other species. Henrich argues that this success is not due to our brain-power, but rather due to our cumulative culture.

*“Probably over a million years ago, members of our evolutionary lineage began learning from each other in such a way that culture became cumulative. … After several generations, this process produced a sufficiently large and complex toolkit of practices and techniques that individuals, relying only on their own ingenuity and personal experience, could not get anywhere close to figuring out over their lifetime. … Once these useful skills and practices began to accumulate and improve over generations, natural selection had to favor individuals who were better cultural learners, who could more effectively tap into and use the ever-expanding body of adaptive information available.” [(* *Henrich, 2016* *), p.3]*

Cumulative cultural knowledge includes technological knowledge like the “know-how” to create arrows or kayaks or compasses, and institutional knowledge like the structure of corporations, churches, and governments. Cultural evolution allows the incremental accumulation of sophisticated designs that could not have been created by any individual during a single lifetime.

In spite of the differences in typical time-scales of biological and cultural evolution, Henrich (Henrich, 2016) provides persuasive examples of gene-culture co-evolution. For example, the cultural acquisition of how-to knowledge about cooking has influenced the biological evolution of the digestive tract in *homo sapiens*. Another example describes how cultural adaptations in tracking and water storage set the context for biological adaptations that have made humans into pre-eminent long-distance runners, able to capture much faster prey by pursuing them to exhaustion.

Some accumulated cultural information is highly adaptive, like the technologies that have allowed humans to inhabit a wider range of environments than any other species on Earth. Others eventually die out, like human sacrifice among the Aztec and Inca, or universal celibacy among the Shakers. The social and individual costs of some cultural beliefs eventually lead to their extinction.

Culture, then, is an evolved adaptation that fills a critical gap in scope and time-scale between biological evolution and individual learning and problem-solving (Richerson and Boyd, 2005). The biological evolution of *Homo sapiens* included the cognitive capacity for shared intentionality (Tomasello, 2019), and social emotions such as shame, guilt, and loyalty (Haidt, 2012).

### 4.3 The Evolution of Social Structures

In *Non-zero: The Logic of Human Destiny* (Wright, 2000), Robert Wright argues that there is a clear direction of progress in human history, visible in the increasing scale of social structures and technologies for supporting cooperation. An organizing theme is the creation of non-zero-sum (i.e., win-win) interactions that result in increasing resources for the society as a whole.

Early humans lived in small egalitarian bands of individuals who cooperated with each other to obtain food through hunting and gathering, and cooperated to protect the band from threats. As the size of human groups increased, egalitarian bands grew into tribes. The successful leader of a tribe, sometimes called a Big Man, was able to accumulate capital and organize the division of labor necessary for building larger-scale technologies such as whale boats and large rabbit nets. Organized hunts using these technologies could bring in much greater resources for the tribe than would be possible even for a very cooperative egalitarian band.

The capture of a whale or many rabbits gives the group a larger supply of perishable meat than it can consume, and therefore an opportunity for trade with other groups–the paradigm win-win interaction. Surplus meat is much more valuable to hungry neighbors who have not had a successful hunt and, in the presence of sufficient trust, can be traded for a commitment to share when circumstances are reversed. Sharing a surplus increases the tribe’s status at relatively low cost, while helping to protect it from future uncertainties. The ability to establish trustworthiness and to recognize and use these forms of cooperation is a selective advantage for a group, which enhances the survival and reproductive opportunities of its individual members.

With new technologies such as agriculture, and increasing scale spanning multiple settlements, tribes grew into chiefdoms. Continued growth, supporting and supported by information technologies such as writing, money, law, and markets, leads to state-level organization: “civilization.” The common link between these information technologies and societal growth is trust. Writing increases trust in promises. Money provides portable, trustworthy value. Published law allows people to trust in the reliability of rules for acceptable behavior. Markets allow trade between people who are willing to trust each other without knowing each other personally. Access to the market motivates people to follow its norms and to punish those who refuse to do so. Increasing scope and benefits of cooperation are supported by political and organizational developments such as democracy, and technological developments such as the industrial revolution(s), the computing revolution, and the Internet.

### 4.4 Taking Stock

Sections 2–4 are intended to support the claim that the human species, consisting of individuals and their societies, is the result of biological and cultural evolution. Biological (genetic) evolution takes place through individual reproductive success. However, individual reproductive success, especially as societies become more complex, depends on the success of the society in accumulating resources including various forms of cultural knowledge.

Cooperation is a large family of mechanisms whereby a society can accumulate more resources. Trust is a relation that is generally necessary for cooperation, both among groups of prospective cooperative partners, and across the entire society in the case of respect for social norms.

Among other roles in human life, the ethics of a society instructs individuals in what it means to be trustworthy, both in one’s own decisions and in recognizing whether others are worthy of trust. Thus ethics (among other things) encourages trust, which encourages cooperation, which helps the society thrive, which helps its individual members thrive, including in terms of individual reproductive success.

My argument in the first half of this essay is that this causal chain contributes to humanity’s success, even up to our very complex modern society. However, the second half of this essay argues that certain links in the chain are vulnerable, and could lead to existential threats.

## 5 Trust and Vulnerability

As we have seen, the definition of trust involves vulnerability among individuals: “*Trust is a psychological state comprising the intention to accept vulnerability based on positive expectations of the intentions or behavior of another.*” [(Rousseau et al., 1998), p.1998].

The vulnerability that individuals accept is vulnerability to cooperative partners (trusting that partners will respect and protect each others’ vulnerabilities, resulting in greater benefits for everyone), and the vulnerability of following social norms (incurring opportunity costs, in confidence that others will do the same, resulting in regularities that make planning easier and reduce the need for defense and repair, for everyone).

In both of these cases, accepting vulnerability by trusting others can result (if the others are trustworthy) in a significantly better outcome than actively defending the vulnerability against exploitation. We can therefore consider trust and cooperation to represent “non-obvious self-interest”, obtaining payoffs from cooperation though prudent acceptance of vulnerability to trustworthy partners.

As described in Figure 1, trust plays a central role in many cooperative processes, ranging from pairs, to larger groups of partners, to the entire society (for social norms). These processes generate the resources that help a society thrive by defending against threats, taking advantage of opportunities, and generally providing benefits for its individual members.

Loss of trust decreases willingness to cooperate and confidence in social norms, resulting in scarcity of resources, making it difficult for the society to plan, and to meet threats or exploit opportunities. Given the centrality of trust in Figure 1, if trust is eroded, society is threatened.

The larger and more complex societies get, the more they rely on trust–of individuals, of institutions, and of social norms (Luhmann, 1979; Wright, 2000). Erosion of trust and loss of resources can bring a successful, complex society to the point of collapse (Tainter, 1988; Diamond, 2005). For our own society, climate change poses an existential threat. Meeting that threat will require serious amounts of trust and cooperation, at a time when trust is being eroded.

## 6 Reasoning With Models

Before returning to the problem of existential threats, we need to consider how we make predictions and action decisions in a world that is essentially infinitely complex. Neither we humans, nor any conceivable computing device, can reason with the full complexity of the physical and social world we inhabit.

Instead, we (ordinary people using common sense as well as scientists and engineers) reason and make decisions using *models* that identify a limited set of relevant factors. We treat all other factors as *negligible*. When the relevant factors are well chosen, a simplified model can efficiently draw conclusions, making predictions, plans, and action decisions that are adequate for the purpose of the model.

The big question of model-building is which few aspects of the unbounded complexity of the world should be explicitly included in the model, omitting everything else. For inference to be feasible, a model must have a small number of elements (variables and constraints in the case of a numerical, algebraic, or differential equation model; constants, variables, relations, and sentences in case of a logical theory; other elements for other types of models). Everything else is left out.

A model makes explicit a relatively small set of “*known unknowns*”—the elements that are relevant to its predictions. The values of some of these known unknowns must be found and provided as inputs; others are derived by inference within the model. The many other aspects of the world not explicitly described in the model are the “*unknown unknowns.*”^3^ For a well-constructed model, omitting the unknown unknowns simply makes the model more efficient.

### 6.1 Deciding What to Do: Game Theory

How do we decide what to do in complex situations with multiple motivated decision-makers and uncertain outcomes? Inspired by recreational games, *game theory* is a powerful framework for creating simple models of these complex situations and interactions (Leyton-Brown and Shoham, 2008; von Neumann and Morgenstern, 1953).^4^ The core idea behind game theory is that each player selects the action that maximizes his own *expected utility*, recognizing that the other players are doing the same. In their seminal book defining game theory [(von Neumann and Morgenstern, 1953), sect.3], von Neumann and Morgenstern show that for any consistent set of preferences that an agent might have over states in the state space, there is a real-valued utility function such that the ordering of its values expresses the agent’s preferences. Unfortunately, we do not have a guarantee that this function is the same as the one provided in the problem statement.

In game theory, action selection by utility maximization is defined as “*rational*.” As in economics and other disciplines, the leading textbook in Artificial Intelligence states that “a rational agent should choose the action that maximizes the agent’s expected utility” [(Russell and Norvig, 2010), p.611].

With a good model, including an appropriate utility measure, game theory can find optimal strategies responding to complex situations, including the optimal choices of other players. Game theory can be effective in real-world circumstances where the stakes and the relationships among the participants are clear–for example in economic interactions such as auctions.

For many decision problems, the game theory models–state and action spaces, transition probabilities, and utility measures–seem to be clear and straight-forward translations of the problem statement. Applying the power of game theory seems to be a matter of plugging in the relevant values, computing expected values, and identifying the maximum. Is this correct?

**FIGURE 3:**
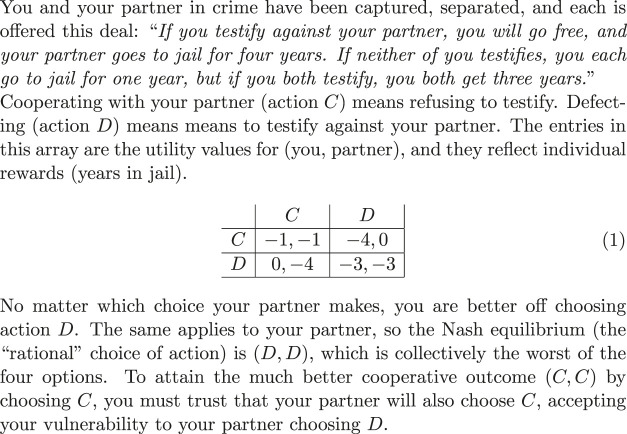
The Prisoner’s dilemma (Axelrod, 1984).

Unfortunately, bad models lead to bad results. The Prisoner’s Dilemma (Figure 3) is famous because, using the straight-forward model, utility maximization gives a poor outcome. A number of other laboratory-scale games provide closely related results, including the Public Goods Game (Rand and Nowak, 2013), the Ultimatum Game (Thaler, 1988), and the Tragedy of the Commons (Hardin, 1968).

**FIGURE 4:**
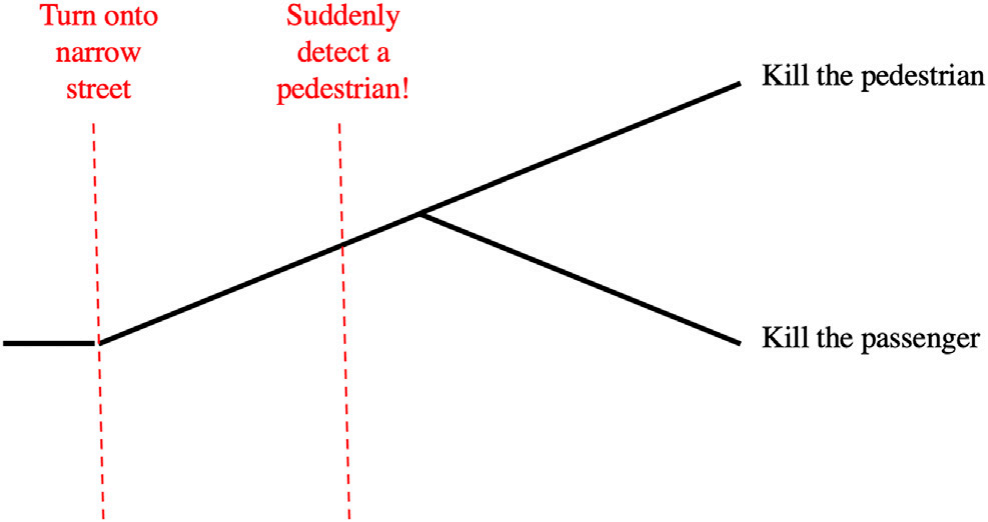
The Deadly Dilemma (abstracted from Awad et al., 2018).

Generalizing the Trolley Problem, a Deadly Dilemma (Figure 4) occurs when an agent is faced with two deadly alternatives. The Moral Machine online survey experiment (Awad et al., 2018) probes the nature of the utility function by which the agent selects the lesser of the two evils. Human participants are shown simulated scenarios where several passengers in an autonomous vehicle are speeding toward several pedestrians on a narrow street. Its only options are to hit the pedestrians, killing all of them, or to crash into a barrier, killing all the passengers. Participants are given demographic features of the potential victims and are asked which choice the AV should make. Assuming that participants are maximizing expected utility, the researchers infer the utilities they assign to those demographic features.

## 7 The Dangers of Bad Models

A good model provides a simplified description of the complex world that can be used efficiently to accomplish the purpose of the model. On the other hand, a bad model can make seriously wrong predictions with unwarranted confidence, failing to predict genuine threats or overlooking genuine opportunities. Particularly dangerous cases occur when the model’s predictions are mostly correct, earning the user’s confidence, but the model is blind to unusual situations where its predictions diverge strongly from reality.

### 7.1 The Problem of Unknown Unknowns

An important failure mode for a model is to omit a factor that proves to be important. This is the infamous “unknown unknown”—a factor missing from the model whose absence is not even suspected, but that leads to an importantly incorrect prediction.

You and your partner in crime have been captured, separated, and each is offered this deal: “*If you testify against your partner, you will go free, and your partner goes to jail for 4 years. If neither of you testifies, you each go to jail for 1 year, but if you both testify, you both get 3 years.*”

Cooperating with your partner (action *C*) means refusing to testify. Defecting (action *D*) means to testify against your partner. The entries in this array are the utility values for (you, partner), and they reflect individual rewards (years in jail).


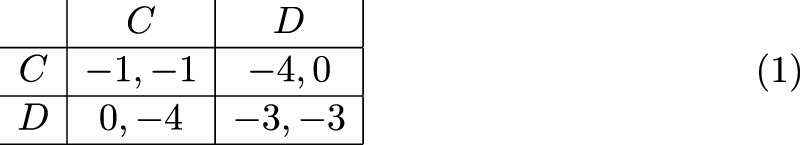


No matter which choice your partner makes, you are better off choosing action *D*. The same applies to your partner, so the Nash equilibrium (the “rational” choice of action) is (*D*, *D*), which is collectively the worst of the four options. To attain the much better cooperative outcome (*C*, *C*) by choosing *C*, you must trust that your partner will also choose *C*, accepting your vulnerability to your partner choosing *D*.

This can be due to modeling error: without thinking about it, the model-builder omits a factor that turns out to be important. For example, in a model of health-care services, an insurance company used cost of treatment as a proxy for severity of disease, failing to recognize that the training data reflected historical racial biases, where minority patients received less (and less costly) treatment for a given severity of disease. For the same clinical evidence, the resulting model categorized diseases as less severe in minority patients than in majority patients (Obermeyer et al., 2019).

This failure mode can also arise when a model that works well in one regime is applied outside that regime, where a simplifying assumption is no longer valid. For example, the effect of air resistance is negligible in a model to predict the result of jumping from my garage roof. But if I consider jumping from a flying airplane, the model *must* include air resistance, or it will be unable to predict the benefit of a parachute.

### 7.2 A Bad Model of the Prisoner’s Dilemma

As the Prisoner’s Dilemma (Figure 3) is presented, the translation from story problem to game theory model seems straight-forward. The choice of action is obvious: Cooperate or Defect. The utility measure is obvious: number of years in prison. Utility maximization clearly shows that Defect is the best choice for each player, no matter what the other player chooses. Shockingly, the outcome (*D*, *D*) from this choice is the *worst* collective result.

More sophisticated games show that this problem generalizes to larger numbers of players (the Public Goods Game (Rand and Nowak, 2013)) and management of limited resources (the Tragedy of the Commons (Hardin, 1968)).

The far better cooperative result (*C*, *C*) is available if each player trusts the other, accepting vulnerability to the other’s defection. However, game theory assumes that each player chooses actions to maximize its own utility measure (as in recreational games). And trust and trustworthiness are unknown, with no role in the utility measure for this model.

If we change the model, adding a component to the utility measure that reflects the player’s demonstrated trustworthiness (say, +1 for *C*, −1 for *D*), then the payoff matrix (1) changes


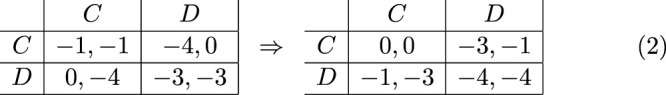


and the best choice for each player is *C*, regardless of the other player’s choice, so utility maximization within this improved model gives the optimal outcome (*C*, *C*).

A reader might argue that the updated payoff matrix (2) no longer represents the Prisoner’s Dilemma, but this is exactly the point. When the payoff from the utility-maximizing choice is obviously worse than available alternatives, then the model of the decision is likely to be wrong. An unknown unknown (in this case trustworthiness) has been omitted from the model. Changing the model improves the outcome.

Much effort has gone into designing models like the Repeated Prisoner’s Dilemma (Axelrod, 1984), aiming to explain how maximizing the expected utility of the future stream of rewards can make the cooperative choice (*C*, *C*) into the optimum. Unfortunately, these efforts have proved to be fragile, for example giving different results for finite and infinite sequences of games, and depending for tractability on repeating the same game. Getting robust decisions for cooperation seems to require trustworthiness to be an explicit element of the model, included in the utility function.

Some critics argue that a game theory model should include only “objective” utilities such as money, mortality, or jail time, rather than “character” or reputation attributes such as trustworthiness. However, note that in a game like poker, expertise clearly includes the ability to estimate an opponent’s character, such as willingness and ability to bluff. A game theory model whose utility measure is based only on the expected values of given hands of cards will play poor poker. The need to estimate trustworthiness in potential cooperative partners is analogous.

In general, an overly simple utility measure will treat important concerns as negligible, leading to a bad outcome.

### 7.3 A Bad Model of the Deadly Dilemma

Likewise, as the Deadly Dilemma (Figure 4) is described, the choice of action is obvious: kill the pedestrians or kill the passengers. The utility measure also seems obvious, especially in the light of the demographic information provided: quality and quantity of lost life (not just number of deaths). As presented, the scenario begins when the autonomous vehicle first senses the pedestrians in its path, and recognizes that its speed and the constrained environment requires it to choose to kill the pedestrians or to kill the passengers. Both alternatives are terrible, so the decision-maker must select the lesser of two evils.^5^

**FIGURE 5:**
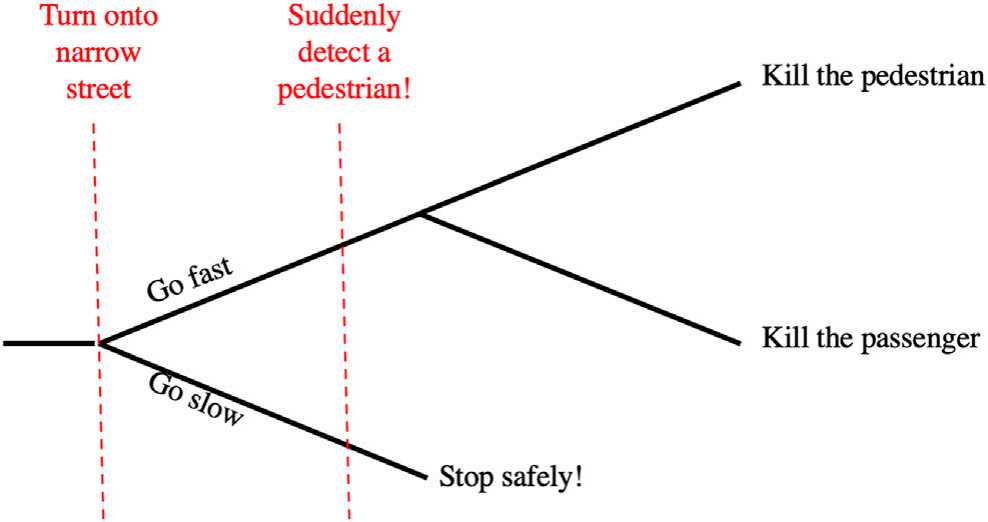
Identify an upstream decision point to avoid the Deadly Dilemma.

In our society, however, beginning drivers are taught situational awareness: continually monitoring the environment and evaluating whether their speed allows them to respond appropriately to sudden developments.^6^ A better model for this decision would include the “upstream decision point” where the environment changes (e.g., the road narrows and loses shoulders), making the vehicle’s speed excessive. At that point, the utility-maximizing decision is to slow down to preserve the ability to make a safe emergency stop in the future, in case a hazard is detected (See Figure 5).

Suddenly facing a deadly dilemma, an individual driver (human or AV) cannot go back in time to the upstream decision point. But an educator or an algorithm designer has the responsibility to anticipate such problems, and ensure that the driver has the situational awareness to detect the upstream decision point and make the choice that avoids the deadly dilemma.

Some readers may argue that a Deadly Dilemma is possible, no matter how unlikely, so an autonomous vehicle should be programmed to make the “right choice” if that should happen. At the point when such a tragic dilemma appears, there is no good option; there is only the lesser of two serious evils. Only in the larger model including the upstream decision point is there an opportunity to make a choice resulting in a good utility value. Therefore, the “moral” choice for the design of an autonomous vehicle is to be prepared for a Deadly Dilemma, use the larger model, recognize the upstream decision point, and choose the option that avoids both evils.

### 7.4 Bad Models Can Target the Vulnerability of Trust

In the Prisoner’s Dilemma, the desirable payoff of the cooperative solution depends on each player trusting the other: accepting vulnerability to defection, confident in the other’s choice to cooperate. In the original model (1), with no utility for trustworthiness, each player is tempted by the even higher payoff from defecting on a trusting partner. However, the symmetry of the game means that the tempting outcome is lost, and both “rational” players do poorly.

When a player’s trust is violated, the victim’s trust for the exploiter is lost, and can only be restored slowly, if at all. Even worse, a reputation for being untrustworthy means that the exploiter will be offered fewer opportunities for cooperation in the future. These are among the reasons why ethical and trustworthy behavior can be considered “non-obvious self-interest.”

Moving beyond the individual to the society, a widespread belief or custom that encourages exploitation results in widespread loss of trust, discouraging cooperation, leading to a tragedy of the commons (Hardin, 1968). This concern has become mainstream, illustrated by a recent discussion in *CACM*, the flagship journal of the Computer Science professional association, of the need for regulating false and polarizing posts on social media platforms.

*“Yet moral hazard may not be a strong enough term to describe what could happen. … another motivation for platform businesses to self-regulate more aggressively is the potential for a “tragedy of the commons.” This phrase refers to a situation where individuals or organizations narrowly pursue their own self-interest, as with moral hazard, but in the process deplete an essential common resource that enabled their prosperity to begin with. Think of the native on Easter Island who cut down the last tree from a once-bountiful forest to make a fire—and then left everyone with an island that had no more trees. With online platforms, we can view the essential common resource as user trust in a relatively open Internet that has become a global foundation for digital commerce and information exchange.” [(* *Cusumano, 2021* *), p.17]*

The erosion of trust can quite possibly lead, not just to economic loss for the exploiters, but to an existential threat to the society as a whole.

## 8 Existential Threats to Human Society?

Our society has grown enormously in size, wealth, complexity, and quality of life over centuries (Pinker, 2018) and millenia (Wright, 2000), due in part to our ability as humans to trust and cooperate with each other, producing net gains in resources for the society as a whole. However, growth and prosperity are not inevitable. Indeed, a number of complex, thriving societies have gone on to collapse due to factors such as overpopulation and ecological disaster (Tainter, 1988; Diamond, 2005).^7^

Decreasing resources can make it more difficult for the society to respond to threats or to take advantage of opportunities. Challenges that were manageable in the past might become insurmountable.

The high-level description in Figure 1 of the roles of ethics, trust, and cooperation in generating society’s resources suggests that trust could be a critical point of vulnerability for a society. A general societal failure of trust could decrease effective cooperation, decreasing available resources.

Are there potential existential threats to our society? Yes, several.

### 8.1 Superintelligent AI

There are concerns about the possibility of an “intelligence explosion” leading to the emergence of an uncontrollable super-intelligent AI that could be an existential threat to humanity. The intelligence explosion was initially proposed by mathematician I. J. Good in 1965 (Good et al., 1965), and explored by computer scientist Vernor Vinge in 1993 (Vinge, 1993), philosopher Nick Bostrom in 2014 (Bostrom, 2014), and computer scientist Stuart Russell in 2019 (Russell, 2019), among many others. Since artificial intelligence today is a product of human intelligence, attaining human-level AI could enable an exponentially self-improving process, possibly resulting in an artificial entity with super-human powers, incomprehensible and uncontrollable by mere humans. Humanity could be eliminated deliberately or by accident. Compelling analogies are presented to the slow rise and sudden take-off of exponential growth curves. Less attention is paid to competing analogies with equally fundamental mathematical phenomena such as the damping effects of resource constraints, and limits to prediction due to sensitive dependence on initial conditions.

Both fictional and non-fictional explorations of this scenario suggest that the existential threat is not actually “super-intelligence” but rather “super-power.” That is, the existential threat follows from putting an AI system with decidedly sub-human levels of intelligence in control of a source of power that poses an existential threat, such as nuclear weapons.

### 8.2 Oversimplified Capitalism

In its abstract ideal form, capitalism is a powerful form of societal cooperation, harnessing feedback cycles among many production and consumption decisions to allocate investment, produce wealth, and distribute that wealth among stakeholding members of society.

The original insight was that, *under appropriate conditions*, a successful economic system need not depend on central coordination to maximize everyone’s utility. As Adam Smith wrote in 1776:

*“It is not from the benevolence of the butcher, the brewer, or the baker that we expect our dinner, but from their regard to their own interest. We address ourselves, not to their humanity but to their self-love, and never talk to them of our necessities but of their advantages.” (* *Smith, 1776* *)*

Those appropriate conditions include market-based competition among many buyers and sellers, both concerned with both quality and price, and all participants being small relative to the size of the market. When this simplified model is appropriate, negative feedback from producer and consumer choices drives the system as a whole toward equilibrium states that satisfy certain optimality critera.

One failure mode for this model occurs when a seller (e.g., of a necessary product) or a buyer (e.g., an employer buying work) dominates their part of the market, to the point where negative feedback can no longer compel them to change their ways. This can easily result in high monopoly prices and low captive-worker wages. Marketplace rules are intended to prevent these possibilities, but a sufficiently powerful player may find it more profitable to manipulate the rules than to improve their offering in the marketplace.

In an influential 1970 article (Friedman, 1970) titled “*The social responsibility of business is to increase its profits*”, the economist Milton Friedman argued that firms in a marketplace should focus purely on profits, without concern for other societal factors. Even when concern for the local community is in the firm’s long-term best interest, Friedman criticized action on that concern as “hypocrisy.” The fictional character Gordon Gecko in the 1987 movie *Wall Street* expressed Friedman’s position with his famous line, “*Greed … is good!*” The reader should be reminded of the oversimplified Prisoner’s Dilemma model (1) with a utility measure sensitive to years in jail but not to trustworthiness, leading to poor decisions with bad outcomes.

These ideas, treating non-financial aspects of the economy (e.g., trust) as negligible, spread from economics and business to the culture generally. Former President Ronald Reagan’s 1986 quote, “*The nine most terrifying words in the English language are: ‘I’m from the government, and I’m here to help’*,” is an explicit attack on the trustworthiness of government (Andersen, 2020).

Well-regulated capitalism is a valuable tool for cooperative enterprise in society. But explicitly discouraging trust also discourages cooperation, reducing resources and threatening the long-term viability of the society.

### 8.3 Climate Change

Climate change is an existential threat to human society, and possibly even to the human species. We’ve passed the “upstream decision point” where a genuine solution might have been possible, but mitigating the destructive impact of climate change will require substantial cooperation among individuals and nations. That cooperation will require trust, which involves vulnerability. Given the global set of actors involved, it is safe to assume that vulnerability will be exploited in some cases. To avoid catastrophe, we will need resources, including trust and cooperation. Can we do it? Nobody knows (Robinson, 2020; Gates, 2021; Kolbert, 2021).^8^

## 9 Conclusion

As an AI researcher, I am concerned about the potential impact of artificially intelligent systems on humanity. The focus of my research has been on understanding the structure of knowledge in commonsense foundational domains (space, dynamical change, objects, actions, and now, ethics), including how this knowledge is created, how it is learned, and how it might be applied to solve tangible problems facing intelligent agents in a complex world.

In the first half of this essay, I present an argument, based on work by Tomasello (Tomasello, 2019), Richerson and Boyd (Richerson and Boyd, 2005), Henrich (Henrich, 2016), Curry (Curry et al., 2016; Curry et al., 2019), Buchanan (Buchanan, 2020), and others, that ethics is an evolved body of cultural knowledge that serves to encourage individual behavior that promotes the welfare of the society (which in turn promotes the welfare of its individual members). A high-level (and partial) representation of the causal paths involved (Figure 1) suggests that *trust* plays a key role in this process.

In the second half of the essay, I consider whether that key role could be a bottleneck, even a vulnerability, exposing the society to existential threats. This possibility depends on the fact that we (humans, AIs, corporations, and governments) necessarily rely on simplifying models to cope with the unbounded complexity of our physical and social world. Well-formulated models are essential tools. But when important unknown unknowns are omitted, poorly-formulated models can draw dangerously wrong conclusions.

By selecting actions to maximize a utility measure, a well-formuilated game theory model can be a powerful and valuable tool. However, a poorly-formulated game theory model may be uniquely harmful, in cases where the action it recommends deliberately exploits the vulnerability and violates the trust of cooperative partners. Widespread use of such models can erode the overall levels of trust in the society. Cooperation is reduced, resources are constrained, and there is less ability to meet challenges or take advantage of opportunities.

We are experiencing a variety of social, economic, and political forces that promote models that erode trust in our society and its institutions and could result in resource limitations. At the same time, humanity is facing the existential threat of climate change, which will require material resources, as well as trust and cooperation.

This argument about the critical importance of trust is not only relevant to robots and other AI systems, important though they may be. Like robots and AIs, corporate and governmental systems make action decisions based on formal representations of simplified models. Human commonsense inference is also subject to errors due to incorrectly simplified models, but most humans have the capability of detecting and correcting model failures, a capability seldom implemented in AI systems.

## Data Availability

The original contributions presented in this study are included in the article. Further inquiries can be directed to the corresponding author.

## Footnotes

1 Some scholars distinguish between “morality” (meaning personal beliefs) and “ethics” (meaning societal teachings), though occasional writers swap the two meanings. I follow the example of philosopher Peter Railton, who writes “I will be using ‘morality’ and ‘ethics’ (and ‘moral’ and ‘ethical’) interchangeably” [(Railton, 2003), Note 1, p.xx].

2 Consider the lesson of the children’s poem, “*The Blind Men and the Elephant*” (Saxe, 1949). Six men, highly educated but blind, conclude that the elephant must be very much like a wall, or a snake, or a leaf, or a spear, or a tree, or a rope, corresponding to the part that each has experienced, while none grasps the complex whole. Fragmentary truths may be useful and important, but must be recognized as incomplete.

3 A model *builder* may knowingly and deliberately omit an aspect of the world from a model, but that aspect is then unknown to the model itself, and very likely to the model *user*, especially if that model user is an artificially intelligent creature. Inferences with that model treat the missing aspects as invisible–they are “unknown unknowns.”

4 The *state space* describes the situations the game can be in, such as the board position in chess and whose move it is; or in baseball, the scores, team at bat, balls and strikes, and runners on bases. Each state has a set of possible *actions*, and a *probability distribution* over the possible results of taking each action. Each player has a *utility value* for each state in the state space, which represents that player’s preference for that state of the game. A *discount factor* decreases the present value of future rewards exponentially with time. The *expected utility* of an action taken in a state is the probability-weighted average of the discounted utilities of all possible future states following from this action.

5 The Moral Machine experiment (Awad et al., 2018) required participants to make this choice in several different scenarios varying on demographic features for the passengers and pedestrians including number, age, gender, social class, criminality, and species. The researchers used the participants’ choices to determine the utilities assigned to those demographic features as a function of the participants’ demographic features including geographical region. The methodological validity and larger significance of this experiment are quite controversial.

6 A well-known question on written driving tests asks students to choose how to react to a ball bouncing into the street in the path of their car. The correct answer is to anticipate that a child could be following the ball, so the driver should slow or stop.

7 There is controversy about sudden societal “collapse” (McAnany and Yoffee, 2010), but general recognition that societies rise and fall, thrive and deteriorate at various points in their history.

8 Humanity has already faced the existential threat of nuclear weapons, capable of destroying our civilization and possibly our species. Somehow, so far, we have found ways to trust and cooperate well enough to keep this existential threat at bay.
